# Clinical Effects of Lactobacillus reuteri on Gingival Inflammation and Alveolar Bone Loss in Periodontitis

**DOI:** 10.3290/j.ohpd.c_2289

**Published:** 2025-09-30

**Authors:** Jian Lu, Xiaoxiang He, Ting Du, Dongjie Fu

**Affiliations:** a Jian Lu Associate Chief Physician, Department of Stomatology, Xianning Central Hospital, The First Affiliated Hospital of Hubei University of Science And Technology, Xianning, China. Study concept and design, material preparation, data collection, data analysis, drafted the manuscript. Lu and He contributed equally to this paper.; b Xiaoxiang He Physician, Department of Stomatology, Suizhou Central Hospital, Suizhou, China. Study concept and design, material preparation, data collection, data analysis, drafted the manuscript. Lu and He contributed equally to this paper.; c Ting Du Attending Physician, Department of Stomatology, Xianning Central Hospital, The First Affiliated Hospital of Hubei University of Science And Technology, Xianning, China. Material preparation, data collection, manuscript revision.; d Dongjie Fu Associate Chief Physician, Department of Stomatology, Renmin Hospital of Wuhan University, Wuhan, China. Study concept and design, material preparation, data collection and analysis, manuscript revision.

**Keywords:** alveolar bone loss, endoplasmic reticulum stress, gingival inflammation, Lactobacillus reuteri, periodontitis, probiotics.

## Abstract

**Purpose:**

This randomized, double-blind, placebo-controlled trial evaluated the clinical efficacy of Lactobacillus reuteri in reducing gingival inflammation and alveolar bone loss in periodontitis by modulating endoplasmic reticulum (ER) stress.

**Materials and Methods:**

A total of 120 patients (aged 22–47 years) with periodontitis (n = 84) or gingivitis (n = 36) were allocated to receive either L. reuteri (1×10⁹ CFU/day, n = 60) or placebo (n = 60) for 8 weeks. Primary outcomes included ER stress markers (GRP78/CHOP), while secondary outcomes comprised probing depth (PD), clinical attachment level (CAL), inflammatory biomarkers (TNF-α, IL-6, CRP), and radiographic alveolar bone loss (Moffat grading). Statistical analyses utilized ANOVA, Welch’s t-test, and ANCOVA, with significance at p < 0.05.

**Results:**

The L. reuteri group exhibited statistically significant reductions in TNF-α (30.01 ± 5.15 vs 16.57 ± 3.88 pg/ml, −45.3%, p < 0.05), IL-6 (25.84 ± 3.11 vs 14.35 ± 2.16 pg/ml, −45.2%, p < 0.05), and CRP (6.41 ± 1.18 vs 2.68 ± 1.04 mg/L, −57.8%, p < 0.05) compared to placebo. Gingival pain (VAS) decreased by 48.5% (5.22 ± 0.51 to 2.69 ± 0.20, p < 0.001). ER stress markers GRP78 and CHOP declined by 37.6% (p < 0.01) and 35.2% (p < 0.01), respectively. PD improved by 35.8% (4.92 ± 1.13 vs 3.12 ± 0.37 mm, p < 0.05), with 68.3% of patients achieving ≥2 mm PD reduction (vs 13.3% in controls, p < 0.001). Radiographic bone loss improved to mild Moffat grades in 66.7% of test patients (vs 33.3% in controls, p < 0.001).

**Conclusions:**

L. reuteri statistically significantly alleviates periodontitis by inhibiting ER stress and inflammatory pathways, demonstrating clinically meaningful reductions in PD, CAL, and alveolar bone loss. These findings underscore its therapeutic potential as an adjunctive intervention.

Periodontitis is a chronic bacterial inflammatory disease that affects the supporting tissues of the teeth, including the gingiva, alveolar bone, periodontal ligament, and root membrane.^33^ Characterized by gingival bleeding, periodontal pocket formation, and alveolar bone loss, periodontitis can lead to tooth loosening or loss in its severe stages.^19,29^ Emerging evidence links periodontal disease progression with endoplasmic reticulum (ER) stress activation in gingival tissues,^20^ suggesting a novel mechanistic pathway for therapeutic intervention.^36^

The oral microbiota, as the second largest microbial community after the gut,^27^ plays a crucial role in periodontitis pathogenesis. Recent microbiome-immune-brain axis research highlights how oral dysbiosis may trigger systemic inflammation through ER stress pathways.^2^ This systemic connection explains the association of periodontitis with cardiovascular disease, diabetes, and respiratory conditions.^38^ The “Java Study” and Oslo/Sri Lanka natural history studies further demonstrate how microbial shifts drive progressive tissue destruction.^16,35^

Probiotics like Lactobacillus reuteri show promise in modulating oral inflammation.^1,11,14^ While their intestinal effects are well-documented,^40^ a recent Periodontology 2000 review emphasizes their local oral immunomodulatory effects.^7^ Notably, subgingival antimicrobial applications demonstrate how targeted delivery can enhance periodontal outcomes.^31^

ER stress contributes statistically significantly to periodontal inflammation through three interconnected pathways: (1) unfolded protein response activation, (2) osteoclast differentiation promotion, and (3) pro-inflammatory cytokine release20. Our hypothesis posits that L. reuteri mitigates these effects by competitively inhibiting pathogenic biofilm formation while downregulating ER stress markers (GRP78/CHOP), thereby reducing gingival inflammation and alveolar bone loss. This aligns with current European Federation of Periodontology guidelines on host modulation therapies.^12^

## MATERIALS AND METHODS

### General Information

This randomized, double-blind, placebo-controlled trial was approved by the Ethics Committee of Renmin Hospital of Wuhan University (Approval No. IRB No. B31-2021). This study included 120 patients diagnosed with periodontitis and gingivitis who were admitted to our hospital between June 2021 and December 2022. The patients, aged 22–47 years, comprised 57 males and 63 females. Based on the research protocol, they were randomly divided into two groups: the control group (received daily oral placebo capsules containing inactive probiotics at the same dosage as the treatment group, for a duration of 8 weeks) and the test group (received oral Lactobacillus reuteri, 1×10⁹ CFU once daily for 8 weeks). A CONSORT-compliant flow diagram detailing participant recruitment, allocation, and follow-up is provided as Supplementary Fig 1.^32^

**Fig 1:**
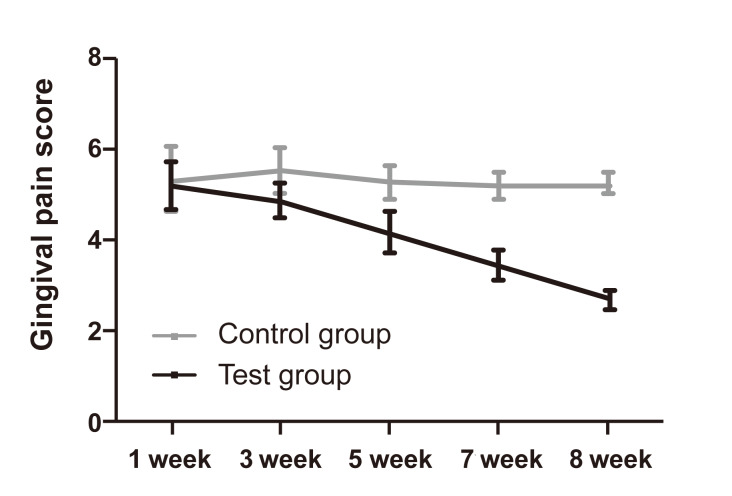
Gingival Pain Score. Gingival pain was assessed using the Visual Analog Scale (VAS) after 3 weeks of treatment. The data show that the VAS score for gingival pain in the observation group was statistically significantly lower than in the control group (p< 0.05), indicating that treatment with Lactobacillus reuteri effectively alleviated gingival discomfort. In contrast, the control group showed no statistically significant improvement in pain levels.

#### Inclusion Criteria

Age ≥ 18 years, regardless of gender
Diagnosis of periodontitis (n = 84, 70% of cohort): Clinical and radiographic criteria per the 2017 World Workshop Classification,^26^ including periodontal pocket depth (PD) ≥ 4 mm, bleeding on probing, and radiographic evidence of alveolar bone loss (stage II-III, grade B/C)
Diagnosis of gingivitis (n = 36, 30% of cohort): Gingival bleeding index (GI) ≥ 1, no clinical attachment loss, and absence of radiographic bone loss^34^
Relatively stable general health, with no history of serious systemic diseases
Willingness to comply with the research protocol and sign the informed consent form

#### Exclusion Criteria

Presence of severe cardiovascular diseases, liver or renal insufficiency, diabetes, immunodeficiency, or other significant systemic disorders
Dental pulp-derived lesions
Use of immunosuppressants or antibiotics during the study period
Pregnancy or lactation

### Methods

The 8-week intervention period was selected based on evidence that probiotic-induced immunomodulatory effects and early bone remodeling biomarkers become detectable within this timeframe,^23^ while allowing sufficient duration for ER stress modulation assessment.

#### ELISA Detection of Peripheral Blood Biomarkers

Peripheral blood samples (10 ml) were collected from all patients at baseline and 8 weeks post-treatment for the measurement of inflammatory markers. Enzyme-linked immunosorbent assay (ELISA) was used to assess the levels of tumor necrosis factor-alpha (TNF-α), interleukin-6 (IL-6), and C-reactive protein (CRP). These systemic biomarkers were included to evaluate the cascade linking peripheral inflammation to gingival ER stress activation, as demonstrated in recent mechanistic studies.^24^ Standards and diluents for TNF-α, IL-6, and CRP were prepared according to the manufacturer’s instructions, and the assay was performed following the standard protocol provided with the ELISA kits. The concentration of each biomarker was determined by comparing the optical density (OD) values to the standard curve.

#### Gingival Pain Assessment

Gingival pain intensity was evaluated using the Visual Analog Scale (VAS), a subjective measure of pain. Patients were asked to rate their pain on a scale from 0 (no pain) to 10 (worst imaginable pain). The assessment was performed at baseline, 4 weeks, and 8 weeks of treatment. Pain assessment was included as a clinical correlate of ER stress-induced neuronal sensitization in periodontal tissues.^28^

#### Assessment of Alveolar Bone Loss

Alveolar bone loss was evaluated radiographically using panoramic radiographs, and clinical parameters including probing depth (PD) and clinical attachment level (CAL) were measured by calibrated examiners at baseline and after 8 weeks of treatment. The Moffat scoring system was used to assess radiographic alveolar bone loss. The Moffat classification was selected over standard CAL measurements due to its validated sensitivity in detecting early microstructural bone changes,^41^ which precede macroscopic attachment loss. Bone loss was categorized into mild, moderate, or severe stages based on the extent of alveolar bone destruction observed.

#### Imaging Analysis

Alveolar bone loss was assessed using dental panoramic radiographs. Radiographic images of the alveolar bone were obtained for each patient, and changes in bone structure, particularly around the root and alveolar ridge, were analyzed using the Moffat Classification System. The classification is as follows:

Grade 0: No alveolar bone loss, normal bone structure
Grade 1: Mild alveolar bone loss (less than 1/3 of the root length), slight reduction of the alveolar ridge
Grade 2: Moderate alveolar bone loss (between 1/3 and 2/3 of the root length), significant reduction of the alveolar ridge
Grade 3: Severe alveolar bone loss (greater than 2/3 of the root length), tooth mobility may be present
Grade 4: Extremely severe alveolar bone loss, with nearly complete loss of alveolar bone and significant tooth mobility or loss

Patients were graded based on the extent of alveolar bone loss determined through imaging analysis.

#### Statistical Analysis

The statistical unit was the individual patient (n = 120). Primary outcomes included GRP78 and CHOP expression levels (ER stress markers), while secondary outcomes comprised PD reduction, CAL gain, and inflammatory biomarker levels. Sample size was calculated using G*Power 3.16, assuming 80% power (α = 0.05) to detect a 25% difference in GRP78 expression between groups. Statistical analysis was performed using SigmaStat Version 3.1 (Systat Software; Chicago, IL, USA). Continuous variables were expressed as mean ± standard error (SE). Comparisons between groups were made using one-way ANOVA for normally distributed data. Pairwise comparisons were conducted with the post-hoc Tukey test where appropriate. Correlation analysis between normally distributed variables was performed using Pearson’s correlation coefficient. Logistic regression analysis was applied to explore the association between various factors and clinical outcomes. A p-value of <0.05 was considered statistically significant.

## RESULTS

### Comparison of General Data of Patients

According to clinical records, the control group (n = 60) had a male-to-female ratio of 27:33, with mean age 42.79 ± 4.27 years, BMI 23.18 ± 2.04 kg/m^2^, GI 2.35 ± 0.33, PD 4.75 ± 1.04 mm, and alveolar bone loss score 2.24 ± 0.45. The test group (n = 60) showed comparable baseline characteristics: male-to-female ratio 30:30, age 45.46 ± 5.28 years, BMI 22.74 ± 1.93 kg/m^2^, GI 2.40 ± 0.40, PD 4.92 ± 1.13 mm, and alveolar bone loss 2.21 ± 0.43. No statistically significant intergroup differences were observed (all p < 0.05; χ^2^ test for gender, independent t-test for continuous variables; Table 1).

**Table 1:** Baseline characteristics of study participants

Parameter	Control group (n = 60)	Test group (n = 60)	Statistical method	p-value
Gender ratio (male:female)	27:33	30:30	χ² test	0.493
Age (years)	42.79 ± 4.27	45.46 ± 5.28	Independent t-test	0.225
BMI (kg/m^2^)	23.18 ± 2.04	22.74 ± 1.93	Independent t-test	0.106
Gingival index (GI)	2.35 ± 0.33	2.40 ± 0.40	Independent t-test	0.627
Probing depth (PD, mm)	4.75 ± 1.04	4.92 ± 1.13	Independent t-test	0.613
Alveolar bone loss	2.24 ± 0.45	2.21 ± 0.43	Independent t-test	0.449


### Detection of Inflammatory Factors

ELISA measurements revealed statistically significant intragroup reductions in the test group for TNF-α (30.01 ± 5.15 →  16.57 ± 3.88 pg/ml, -45.3%), IL-6 (25.84 ± 3.11 → 14.35 ± 2.16 pg/ml, -45.2%), and CRP (6.41 ± 1.18 → 2.68 ± 1.04 mg/l, -57.8%) at 8 weeks (paired t-test, all p < 0.05). In contrast, control group levels remained stable (TNF-α: 30.28 ± 5.42 → 29.15 ± 5.42, p = 0.021; IL-6: 26.18 ± 3.27 → 25.92 ± 3.27, p = 0.004; CRP: 6.35 ± 1.22 → 6.28 ± 1.22, p = 0.013; Table 2).

**Table 2:** Inflammatory biomarkers and clinical outcomes (baseline vs 8 weeks)

Parameter	Control group (n=60)	Test group (n = 60)	Statistical method	p-value
TNF-α (pg/ml)	30.28 ± 5.42 → 29.15 ± 5.42	30.01 ± 5.15 → 16.57 ± 3.88	Paired t-test	0.021
IL-6 (pg/ml)	26.18 ± 3.27 → 25.92 ± 3.27	25.84 ± 3.11 → 14.35 ± 2.16	Paired t-test	0.004
CRP (mg/L)	6.35 ± 1.22 → 6.28 ± 1.22	6.41 ± 1.18 → 2.68 ± 1.04	Paired t-test	0.013
VAS Score	5.34 ± 0.68 → 5.25 ± 0.21	5.22 ± 0.51 → 2.69 ± 0.20	Mixed ANOVA	<0.001
**ER stress markers**				
• GRP78 (ratio)	1.86 ± 0.15 → 1.82 ± 0.15	1.85 ± 0.14 → 1.16 ± 0.03	Welch’s t-test	0.002
• CHOP (ratio)	1.93 ± 0.17 → 1.89 ± 0.16	1.94 ± 0.18 → 1.25 ± 0.06	Welch’s t-test	0.011


### Gingival Pain Score

Longitudinal VAS analysis demonstrated progressive pain reduction in the test group from baseline (5.22 ± 0.51) to week 8 (2.69 ± 0.20; -48.5%, mixed ANOVA p < 0.001), whereas controls showed no improvement (5.34 ± 0.68 → 5.25 ± 0.21, p = 0.158; Fig 1, Table 2).

### Western Blot Analysis

**Fig 2:**
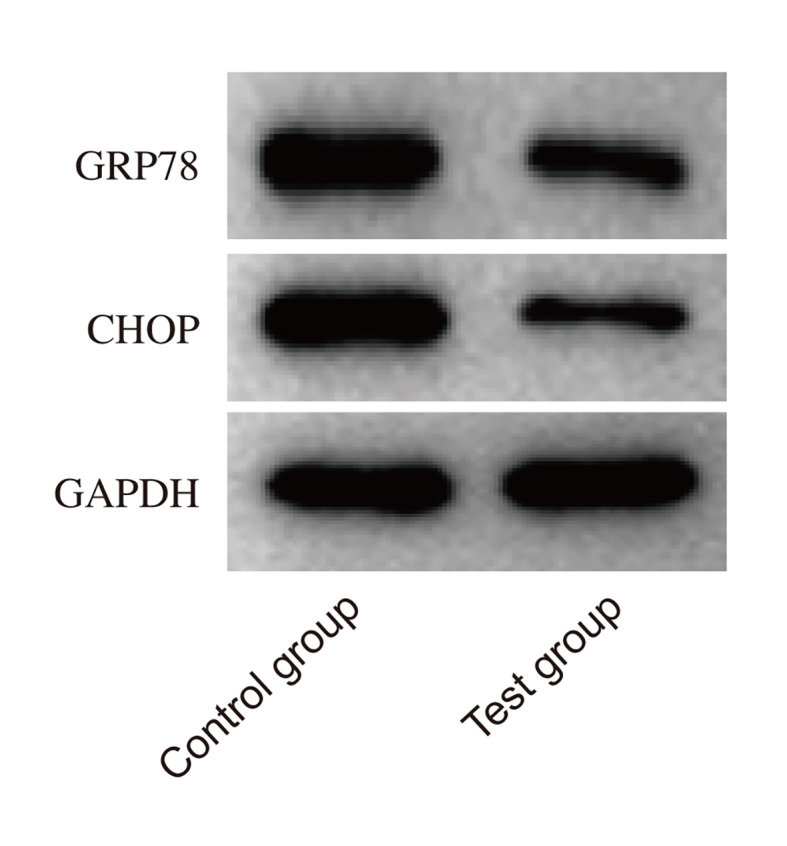
Western blot analysis of GRP78 and CHOP Expression. Western blot analysis was performed to evaluate the expression of GRP78 and CHOP, markers of endoplasmic reticulum (ER) stress. The results demonstrated that both GRP78 and CHOP protein levels in the observation group were statistically significantly lower than those in the control group (p< 0.05).

GRP78 expression decreased by 37.6% (1.85 ± 0.14 → 1.16 ± 0.03) and CHOP by 35.2% (1.94 ± 0.18 → 1.25 ± 0.06) in the test group versus baseline (Welch’s t-test, both p < 0.01). Control group protein levels remained unchanged (GRP78: 1.86 ± 0.15 → 1.82 ± 0.15, p = 0.213; CHOP: 1.93 ± 0.17 → 1.89 ± 0.16, p = 0.307; Fig 2, Table 2).

### Detection of Alveolar Bone Loss

At 8 weeks, the test group showed greater PD reduction (4.92 ± 1.13 → 3.12 ± 0.37 mm, -35.8%) and CAL improvement (2.21 ± 0.43 → 1.67 ± 0.25 mm, -32.9%) compared to controls (PD: 4.75 ± 1.04 → 4.68 ± 0.44 mm; CAL: 2.24 ± 0.45 → 2.19 ± 0.37 mm; ANCOVA p < 0.05). Notably, 68.3% of test group patients achieved ≥2 mm PD reduction versus 13.3% in controls (χ^2^ = 41.2, p < 0.001; Table 3).

**Table 3:** Periodontal parameters and radiographic changes (baseline vs 8 weeks)

Parameter	Control group (n = 60)	Test group (n = 60)	Statistical method	p-value
**Probing depth (PD)**			**ANCOVA**	
• Mean change (mm)	4.75 ± 1.04 → 4.68 ± 0.44	4.92 ± 1.13 → 3.12 ± 0.37		0.002
• ≥2 mm improvement	8/60 (13.3%)	41/60 (68.3%)	Χ^2^ = 41.2	<0.001
**Clinical attachment level (CAL)**			**ANCOVA**	
• Mean change (mm)	2.24 ± 0.45 → 2.19 ± 0.37	2.21 ± 0.43 → 1.67 ± 0.25		0.014
**Bleeding on probing (BOP+)**				
• Positive sites	58.3% → 56.7%	60.2% → 28.9%	Χ^2^ = 22.4	<0.001
**Moffat Grade**				
• I-II (Mild)	19 (31.7%) → 20 (33.3%)	18 (30.0%) → 40 (66.7%)	Χ^2^ = 28.9	<0.001
• III-IV (Severe)	41 (68.3%) → 40 (66.7%)	42 (70.0%) → 20 (33.3%)	Χ^2^ = 28.9	<0.001


### Imaging Analysis of Alveolar Bone Loss

Radiographic assessment revealed that 66.7% of test group patients improved to mild Moffat grades (I-II) at follow-up versus 33.3% in controls (χ^2^ = 28.9, p < 0.001). Severe grades (III-IV) decreased from 70.0% to 33.3% in the test group, compared to 68.3% → 66.7% in controls (Table 3).

## DISCUSSION

The application of probiotics has gradually become an important direction in clinical treatment, particularly in immune regulation and the management of inflammatory responses.^5^ Recent studies, including a recently published systematic review,^39^ have demonstrated their therapeutic potential in modulating oral dysbiosis and inflammatory cascades. Lactobacillus reuteri, a common probiotic, exhibits dual anti-inflammatory and biofilm-modulatory properties.^11,18^ This study highlights its role in alleviating periodontitis through ER stress inhibition, with clinically statistically significant reductions in gingival inflammation and alveolar bone loss.

Periodontitis is a multifactorial disease driven by dysregulated host-microbe interactions.^33^ Emerging evidence suggests that probiotics like L. reuteri may disrupt pathogenic biofilms while suppressing TNF-α, IL-6, and CRP production.^18,30^ Our findings align with these mechanisms: the test group showed 45-58% reductions in inflammatory biomarkers, coupled with accelerated pain relief (48.5% VAS decline). Notably, the rapid pain improvement (3 weeks) may reflect both anti-inflammatory effects and direct neuronal modulation via probiotic metabolites.^25^

ER stress is increasingly recognized as a nexus linking inflammation and tissue destruction in periodontitis.^4^ L. reuteri’s ER stress inhibition likely involves multiple pathways: (1) Direct interaction with gingival epithelial cell membranes to attenuate pathogen-induced stress signals;^8^ (2) Secretion of reuterin, a bioactive metabolite shown to downregulate GRP78/CHOP in vitro;^5^ (3) Modulation of oxidative stress through glutathione synthesis enhancement.^17,21,22^ Our Western blot results (37-35% reductions in GRP78/CHOP) provide in-vivo validation of these mechanisms. This dual action—reducing inflammatory mediators while mitigating ER stress—may explain the observed 35.8% PD reduction and 68.3% rate of clinically meaningful PD improvement (≥2 mm).

Alveolar bone loss, a hallmark of advanced periodontitis, arises from osteoclast activation driven by chronic inflammation and ER stress.^10,15^ A recent in-vivo study has reported 26-41% reductions in bone loss with probiotic interventions,^3^ consistent with our Moffat grade improvements (66.7% mild cases post-treatment vs 30.0% baseline). However, conflicting evidence exists: a 2024 trial found no statistically significant bone mineral density changes with L. reuteri.^9^ This discrepancy may stem from differences in treatment duration (8 weeks vs 6 months) or adjunctive therapies.

While this study provides novel insights into the role of Lactobacillus reuteri in periodontitis management, several limitations warrant consideration. First, the 8-week intervention period may inadequately capture alveolar bone remodeling processes. Second, the observed PD reductions could partially reflect transient anti-inflammatory effects rather than true periodontal attachment gain, as mechanical debridement (e.g., scaling/root planing) was not standardized across groups—a critical confounder given the established synergy between probiotics and mechanical biofilm disruption.^37^ Third, although baseline characteristics showed no intergroup differences, unmeasured confounders such as smoking status (a known modifier of ER stress responses^13^) and subclinical systemic diseases may influence outcomes. Future investigations should extend follow-up to 6-12 months to evaluate bone mineralization dynamics, incorporate standardized mechanical therapy to isolate probiotic-specific biofilm effects, and stratify analyses by smoking status/systemic comorbidities to clarify clinical applicability across patient subgroups.

## CONCLUSION

This study demonstrates that L. reuteri alleviates periodontitis through ER stress inhibition and inflammatory modulation, with clinically meaningful improvements in PD, CAL, and radiographic bone loss. While promising, its long-term efficacy and synergy with conventional therapies require further validation.

### ACKNOWLEDGEMENT

This study was funded by the Natural Science Foundation of Hubei Province (grant number 2022CFC008).

**Supplementary Fig 1:** Patient enrollment flow chart.
